# Recurrent Metatarsal Fractures in a Patient With Cushing Disease: A Case Report

**DOI:** 10.7759/cureus.25015

**Published:** 2022-05-15

**Authors:** Jose Iturregui, Glenn Shi

**Affiliations:** 1 Department of Orthopaedic Surgery, Mayo Clinic, Jacksonville, USA

**Keywords:** cushing disease, cushing syndrome, pituitary adenoma, fragility fracture, metatarsal fracture

## Abstract

Cushing syndrome (CS) can result from excess exposure to exogenous or endogenous glucocorticoids. The most common endogenous cause of CS is an adrenocorticotropic hormone (ACTH)-secreting pituitary adenoma, known as Cushing disease (CD). Patients typically present with characteristics including truncal obesity, moon facies, facial plethora, proximal muscle weakness, easy bruising, and striae. Insufficiency fractures of the metatarsals are a rare presentation for CS. A 39-year-old premenopausal woman presented to the orthopedic outpatient clinic with recurrent metatarsal fractures and no history of trauma. A metabolic bone disease was suspected, and after further evaluation by endocrinology services, the CD was diagnosed. Surgical resection was performed, and pathology confirmed the presence of a pituitary adenoma. Multiple, recurrent, non-traumatic metatarsal fractures can be the initial presentation of CD in a premenopausal woman.

## Introduction

Cushing syndrome (CS) is a rare clinical and metabolic disorder caused by excessive exposure to glucocorticoids. In the United States, an estimated 10 to 15 people per million population are affected by CS each year, while studies in Europe report an incidence of 0.7 to 2.4 per million people affected annually [1,2]. Furthermore, CS more commonly affects women [2]. Common characteristics of CS include truncal obesity, moon facies, proximal muscle weakness, fatigue, facial plethora, supraclavicular fullness, peripheral edema, weight gain, striae, easy bruising, acne, hirsutism, amenorrhea, dorsocervical "buffalo" hump, depression, hypertension, impaired glucose tolerance, and osteoporosis [1,3,4].

The most common cause of CS is exogenous glucocorticoid therapy. Meanwhile, endogenous cortisol hypersecretion commonly results from either an adrenocorticotropic hormone (ACTH)-secreting pituitary adenoma or a cortisol-secreting adrenal tumor. When CS is caused by a pituitary adenoma, this is referred to as Cushing disease (CD). CD is the most common endogenous cause of CS, accounting for 80-85% of cases [1,5].

Whether a patient’s CS is caused by exogenous or endogenous sources, excessive exposure to steroids can have deleterious effects on the bones, resulting in secondary osteoporosis. The decrease in bone mass and microarchitectural changes increase the risk of fragility fractures, with reported rates as high as 30-67% [6]. The most commonly reported fracture site in CS patients is the vertebrae; however, other reported fracture sites include the ribs, sternum, wrist, elbow, shoulder, pelvis, hip, femoral condyles, tibia, fibula, calcaneus, metatarsals, and phalanges [4,6-16]. There are reports of metatarsal fractures occurring in patients diagnosed with endogenous CS [3,6,7,16-19]. However, to the best of our knowledge, there are no reports of multiple, recurrent, bilateral metatarsal fractures as the initial presentation in a pre-menopausal woman with CD. Here, we present a case of a premenopausal woman with recurrent metatarsal stress fractures who was diagnosed with CD after further evaluation.

## Case presentation

**Figure 1:**
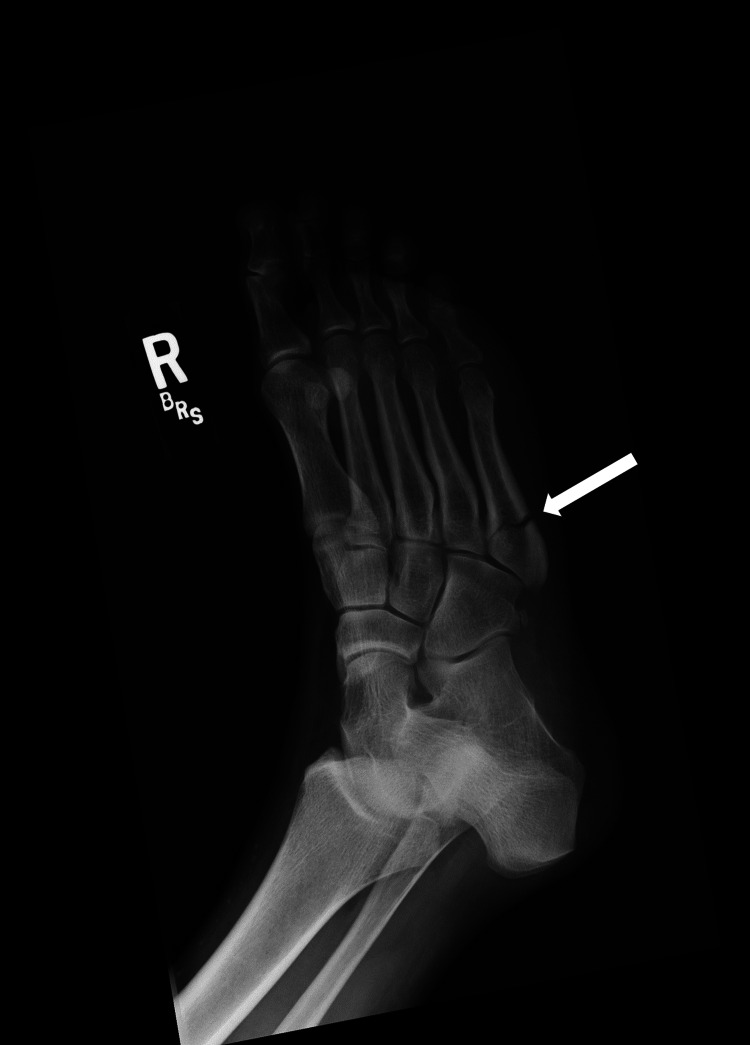
Oblique radiograph of the right foot demonstrating a mildly displaced transverse fracture of the proximal fifth metatarsal (arrow).

A 39-year-old premenopausal woman was evaluated by her primary care physician due to right foot pain after feeling a pop while walking. She reported swelling and some bruising along the lateral aspect of her foot. Her exercise regimen consisted of walking twice a week for 30 minutes at each session. She did not report any traumatic injuries to her foot. Imaging revealed a fifth metatarsal fracture (Figure 1). The patient was placed in a cast walker boot and referred to orthopedics for further evaluation. Orthopedic management included no weight bearing on her right foot and continuing using the cast walker boot or a postop shoe, with reevaluation in four weeks.

At the time of evaluation, the patient was 161.5 cm tall, weighed 101 kg, and had a BMI of 38.86 kg/m^2^. Her medical history included hypertension, hyperglycemia, hyperlipidemia, hypothyroidism, obesity, anxiety, obstructive sleep apnea, and colon polyps. The patient reported a history of metatarsal fractures in her left foot in 2008, which healed slowly and without surgical intervention. She also underwent bunion and bunionette surgery on her left foot. Her medications included alprazolam, levothyroxine, lisinopril, bimatoprost, ergocalciferol, meloxicam, and ondansetron. She was a former smoker (2007-2010), a daily wine drinker, and had an active job working as a nurse. Her family history included lung cancer and alcohol abuse in her father; hypertension, hypothyroidism, and alcohol abuse in her mother; and osteoporosis and end-stage renal disease secondary to polycystic kidney disease in her sister.

**Figure 2:**
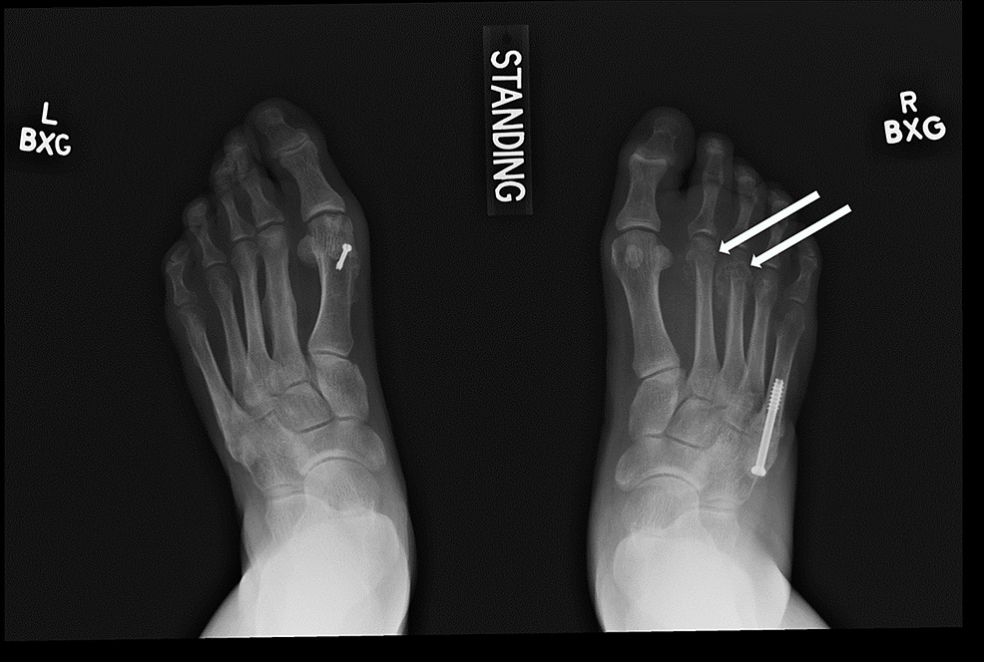
Anteroposterior radiograph of bilateral feet demonstrating second and third metatarsal neck fractures of the right foot (arrows).

At the three-month follow-up visit, the fracture line remained clearly visible, and minimal callus had formed at the fracture site. Surgical fixation was recommended and performed four months after the fracture occurred. Six months after her right foot's fifth metatarsal fracture, she developed new-onset swelling and tenderness over the middle metatarsals dorsally in her right foot with no history of trauma. Radiographs demonstrated new second and third metatarsal neck fractures (Figure 2). Conservative management with a postop shoe for six weeks and re-evaluation was recommended. In the interim between her initial right foot fifth metatarsal fracture and the new right foot second and third metatarsal fractures, the patient was diagnosed with diabetes mellitus type II, treated with a plant-based diet, hospitalized for urolithiasis, and diagnosed with depression. She was started on bupropion. 

Due to the recurrent metatarsal stress fractures with no associated trauma, the patient was referred to endocrinology for workup of metabolic bone disease. Her physical exam revealed no abnormalities, and her overall workup was negative. Bone mineral density results demonstrated osteopenia in the lumbar spine (T-score: -1.8) and left femoral neck (T-score: -1.0), and normal bone density in the left total hip (T-score: -0.80).

**Figure 3:**
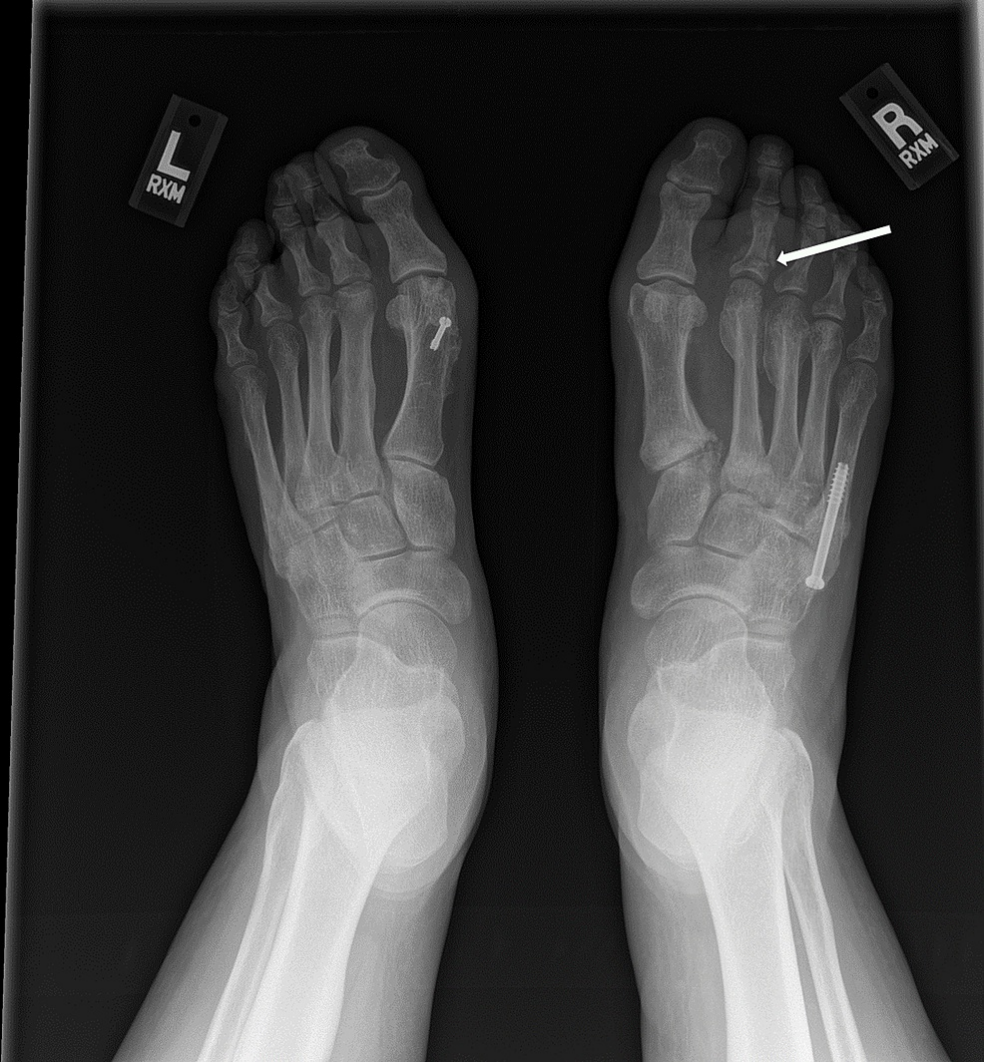
AP radiograph of bilateral feet demonstrating a subacute fracture of the second proximal phalanx of the right foot (arrow).

**Figure 4:**
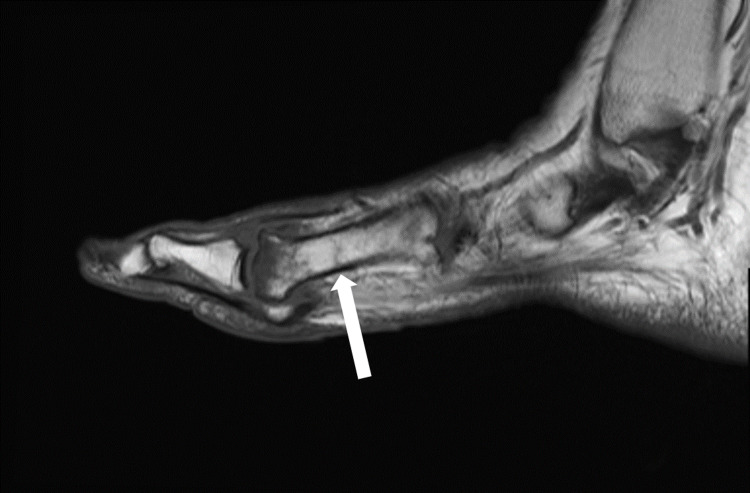
T1-weighted sagittal MRI of the right foot demonstrating a first metatarsal shaft stress fracture (arrow).

Six months following her right foot's second and third metatarsal fractures, the patient developed right great toe and second toe swelling and bruising. Two months later, after trying supportive tennis shoes and reducing weightbearing on her right foot, she did not notice any improvement and sought orthopedic care. Radiographs revealed a new subacute fracture of the right second proximal phalanx (Figure 3). A magnetic resonance imaging (MRI) scan was ordered, which revealed a first metatarsal shaft stress fracture as well (Figure 4). She underwent conservative management with a Cam walker boot and was referred to endocrinology for re-evaluation for suspected endocrinopathy.

**Figure 5:**
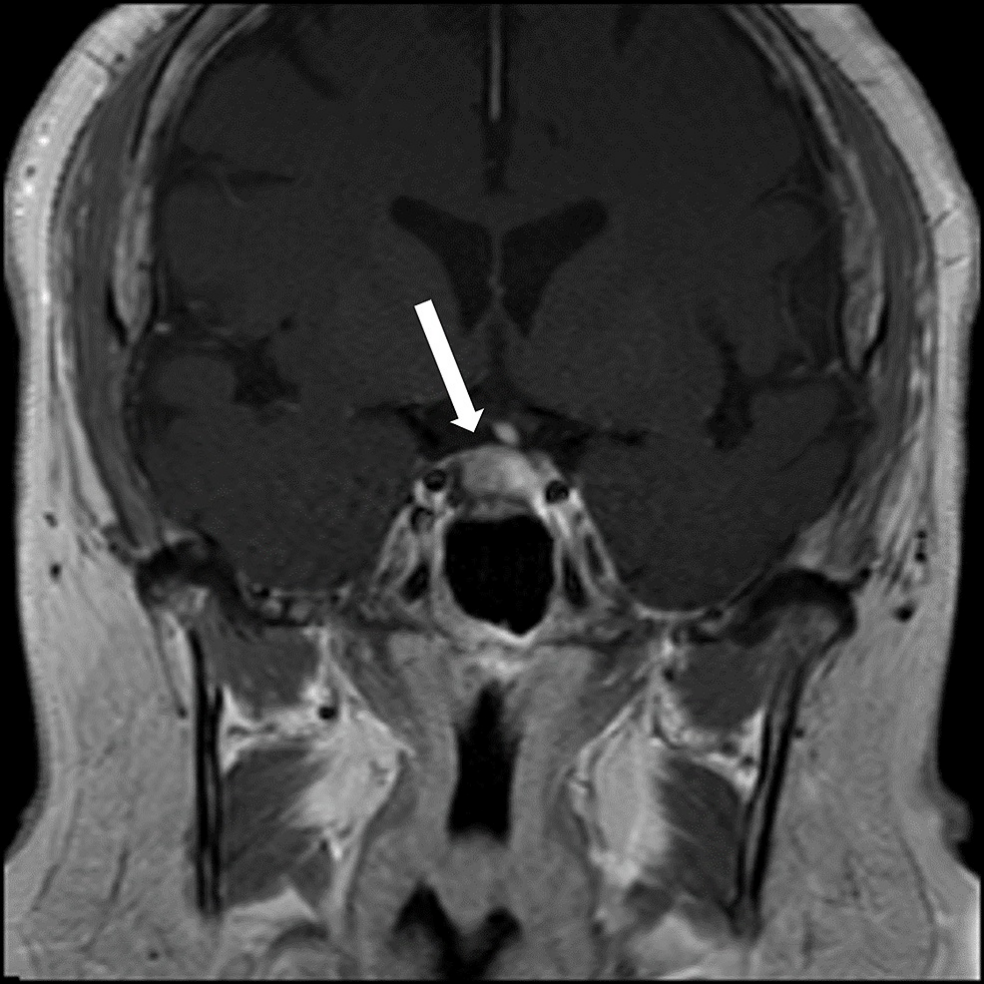
T1-weighted coronal MRI of the pituitary demonstrating a 1.1 cm × 1.5 cm × 1.1 cm cystic sellar mass which represents a pituitary macroadenoma (arrow).

At her endocrinology visit, a physical exam revealed some facial hair, frontal hair loss, and a significant dorsocervical and anterior cervical fat pad. A Cushingoid face shape, facial redness, acne, oligomenorrhea, incremental weight gain over the last decade, centripetal adiposity, easy bruising, and lower leg swelling were also reported. Bone mineral density results reported spine and hip Z-scores within the expected range for age, indicating no osteoporosis. Since she had features of hypercortisolism, labs to evaluate for Cushing syndrome were ordered. The 11:00 pm salivary cortisol levels were elevated to 173 ng/dL and 168 ng/dL in two samples. The 1 mg dexamethasone suppression test failed to suppress her cortisol levels, with an elevated cortisol value of 29 mcg/dL. The 24-hour urine-free cortisol level was elevated at 135 mcg/24 hours. These lab results confirmed a diagnosis of Cushing syndrome. Her ACTH was elevated at 86 pg/mL, which indicated an ACTH-dependent CS. Pituitary MRI demonstrated a 1.1 cm × 1.5 cm × 1.1 cm pituitary lesion, representing a pituitary macroadenoma (Figure 5). The patient underwent endoscopic endonasal transsphenoidal pituitary tumor resection with the goal of treating her Cushing disease and preventing further fragility fractures. Pathology evaluation confirmed a pituitary adenoma.

## Discussion

This is a case of a 39-year-old woman who presented with recurrent metatarsal fractures with no history of trauma, raising suspicion of a metabolic bone disease. The patient also developed centripetal weight gain, glucose intolerance, kidney stones, depression/anxiety, and Cushingoid features. A laboratory workup performed by endocrinology services confirmed a diagnosis of ACTH-dependent CS. An MRI revealed a pituitary lesion which represented a pituitary macroadenoma, for which surgical resection was performed. Pathology confirmed a pituitary adenoma. The association of multiple, non-traumatic metatarsal fractures occurring in premenopausal women with endogenous CS has been reported in the literature [3,7,19]. However, to the best of our knowledge, this is the first report presenting a premenopausal woman with multiple, recurrent metatarsal fractures as the initial manifestation of CD.

Several mechanisms play a role in glucocorticoid-induced bone loss, which is more prominent in trabecular bone compared to cortical bone [3,4,6,8]. Normally, trabecular bone has a greater bone turnover rate than cortical bone. In the presence of excess glucocorticoids, trabecular bone has greater sensitivity to glucocorticoids and undergoes slower bone turnover. The most significant effects of excess glucocorticoids on bones are decreased osteoblast function and quantity, which explain the reduced trabecular bone turnover rate [4,10]. The proposed mechanisms for this are glucocorticoid-induced inhibition of osteoblast proliferation and genesis, as well as induction of osteoblast and osteocyte apoptosis [4,10,11]. Furthermore, glucocorticoids decrease bone protein synthesis (e.g., osteocalcin), type I collagen formation, and alkaline phosphatase activity [4]. Additional effects include greater bone resorption, inhibition of intestinal calcium absorption, inhibition of renal calcium reabsorption, and decreased secretion of gonadal steroids and growth hormones [8]. Glucocorticoids also induce protein catabolism, which can result in muscle weakness, decreased bone stimulation from weakened muscle contraction, and further bone loss and debility [4].

Multiple fragility fractures in the foot with no history of trauma or overuse are uncommon. When evaluating a patient with this presentation, secondary causes for these fractures need to be investigated. Differential diagnoses include osteoporosis, Charcot foot, multiple myeloma, celiac disease, avascular necrosis, and endocrine disorders such as hyperthyroidism, primary hyperparathyroidism, or CS, among others [3,6,7].

There is a high rate of fragility fractures due to secondary osteoporosis in CS patients, with the vertebrae being most commonly affected [6]. LiYeung and Lui [7] and Albon et al. [19] each reported a case of a pre-menopausal woman who initially presented with multiple metatarsal fractures secondary to an adrenal adenoma causing CS. In each case, the patient’s densitometry indicated osteoporosis. However, in our case and the case reported by Molnar et al. [3] of a pre-menopausal woman with multiple fractures due to CD (recurrent fractures were not reported), the bone densitometries performed did not indicate osteoporosis.

The patients reported by LiYeung and Lui [7], Albon et al. [19], and Molnar et al. [3] did not demonstrate marked clinical characteristics of CS. In comparison to our patient, she did have multiple Cushingoid features upon her second evaluation by endocrinology. Furthermore, in all our cases, the patients were first evaluated for metatarsal fractures as the initial presentation, which resulted in a diagnosis of endogenous CS after further evaluation.

Finally, early recognition and treatment of CS are important, as there is an increased risk of morbidity and mortality as the condition progresses [20]. In addition, the treatment of CS can reverse the bone loss that occurs with excess glucocorticoid exposure [4,10]. This case also highlights the importance of collaboration between physicians in the different branches of medicine.

## Conclusions

Excess glucocorticoid exposure can have deleterious effects on the bones, increasing the risk for secondary osteoporosis and fragility fractures. There needs to be an index of suspicion for metabolic bone disease, including endogenous CS caused by CD, as the underlying etiology of multiple, recurrent, atraumatic metatarsal fractures in pre-menopausal women. Early diagnosis and management of CD can lower the risk of morbidity and mortality as well as reverse bone loss.

## References

[1] Guaraldi F, Salvatori R (2012). Cushing syndrome: maybe not so uncommon of an endocrine disease. J Am Board Fam Med.

[2] Valassi E, Santos A, Yaneva M (2011). The European Registry on Cushing's syndrome: 2-year experience. Baseline demographic and clinical characteristics. Eur J Endocrinol.

[3] Molnar V, Zekan P, Dušek T, Ivković A (2021). Multiple metatarsal fractures: the first manifestation of Cushing’s disease—a case report. J Am Podiatr Med Assoc.

[4] Han JY, Lee J, Kim GE (2012). A case of cushing syndrome diagnosed by recurrent pathologic fractures in a young woman. J Bone Metab.

[5] Barahona MJ, Sucunza N, Resmini E (2009). Deleterious effects of glucocorticoid replacement on bone in women after long-term remission of Cushing's syndrome. J Bone Miner Res.

[6] Papadakis G, Uebelhart B, Goumaz M, Zawadynski S, Rizzoli R (2014). An unusual case of hypercortisolism with multiple weight-bearing bone fractures. Clin Cases Miner Bone Metab.

[7] LiYeung LL, Lui TH (2015). Bilateral adrenal adenoma presented as multiple metatarsal and phalangeal fractures. J Orthop Case Rep.

[8] Trementino L, Appolloni G, Ceccoli L, Marcelli G, Concettoni C, Boscaro M, Arnaldi G (2014). Bone complications in patients with Cushing's syndrome: looking for clinical, biochemical, and genetic determinants. Osteoporos Int.

[9] Abdel-Kader N, Cardiel MH, Navarro Compan V, Piedra Priego J, González A (2012). Cushing's disease as a cause of severe osteoporosis: a clinical challenge. Reumatol Clin.

[10] Lee HJ, Je JH, Seo JH, Na YJ, Yoo HJ (2014). Multiple spontaneous rib fractures in patient with Cushing’s syndrome. J Bone Metab.

[11] Poonuru S, Findling JW, Shaker JL (2016). Lower extremity insufficiency fractures: an underappreciated manifestation of endogenous Cushing's syndrome. Osteoporos Int.

[12] Belaya ZE, Hans D, Rozhinskaya LY (2015). The risk factors for fractures and trabecular bone-score value in patients with endogenous Cushing's syndrome. Arch Osteoporos.

[13] Tajika T, Shinozaki T, Watanabe H, Yangawa T, Takagishi K (2002). Case report of a Cushing's syndrome patient with multiple pathologic fractures during pregnancy. J Orthop Sci.

[14] Baron E, Sheinfeld M, Migdal EA, Hardoff R (1996). Multiple pathologic fractures mimicking bone metastases in a patient with Cushing's syndrome. Clin Nucl Med.

[15] Bosch S, Bogaerts S (2021). Pituitary adenoma presenting with bilateral calcaneal stress fracture: a case report. JOSPT Cases.

[16] Kostoglou-Athanassiou I, Spiliotis G, Athanassiou L, Myriokefalitakis I (2018). Cushing’s syndrome in a patient with systemic lupus erythematosus. Endocrine Abstracts.

[17] Kaur K, Findling JW (2008). Cushing’s disease. A Case-Based Guide to Clinical Endocrinology.

[18] Ontell FK, Shelton DK (1995). Multiple stress fractures. An unusual presentation of Cushing's disease. West J Med.

[19] Albon L, Rippin J, Franklyn J (2003). “My feet are killing me!” An unusual presentation of Cushing’s syndrome. Endocrine Abstracts.

[20] Nieman LK (2018). Recent updates on the diagnosis and management of Cushing’s syndrome. Endocrinol Metab (Seoul).

